# Personal

**Published:** 1901-01

**Authors:** 


### PERSONAL.

RECENT army orders announced the appointment of Maj. Ira S. Brown, surgeon, U. S. V., to be chief surgeon of the district of Panay, Philippine Islands. This appointment is in the nature of a promotion in recognition of Dr. Brown's efficient service.

DR. JOHN T. PITKIN, of Buffalo, read a paper on How to Print the *x*-Ray Picture for Details, before the Röntgen Society during its recent meeting at New York.

DR. WILLIAM WARREN POTTER has been appointed a member of the auxiliary committee of the third Pan-American Medical Congress, to be held at Havana, February 5-9, 1901.

DR. J. HENRY CARSTENS, of Detroit, Mich, by invitation addressed the Marion County Medical Society, at Indianapolis, Ind., November 13, 1900, on the subject of The Diagnosis and Treatment of Gallstones. Dr. Carstens gave an operative clinic at the City Hospital, and was entertained at dinner by Dr. L. H. Dunning.

DR. VICTOR C. VAUGHAN, of Ann Arbor, Mich., addressed the alumni and students of the University of Buffalo, Friday afternoon, December 21, 1900, at 4.30 o'clock. The subject of his discourse was Bacterial Toxins.

DR. JAMES T. JELKS, of Hot Springs, Ark., the accomplished editor of the *Hot Springs Medical Journal*, delivered the dedicatory address at the opening ceremonies of the new and commodious infirmary recently established by the Sisters of Charity at Little Rock, Ark.

Dr. Edward B. Angell, of Rochester, visited Buffalo on Monday, December 17, 1900, to attend the meeting of the Western New York Alumni Association of the University of Pennsylvania. Dr. Angell was chosen president of the Association.

---

Dr. Roswell Park and Dr. Charles Cary, of Buffalo, recently visited Mexico as the guest of Mr. Frank H. Goodyear, who entertained a group of friends in his private car. The tourists were absent about three weeks, returning to Buffalo in season for the Christmas holidays.

---

Dr. William Ridgeley Stone, of New York, visited Buffalo on Tuesday, November 27, 1900, and read a paper before the section on obstetrics at the Buffalo Academy of Medicine, entitled Cocainisation of the spinal cord in Labor by means of Lumbar Puncture. Dr. Stone was entertained at luncheon by Dr. Albert J. Colton, chairman of the section.

---

Dr. Chauncey Pelton Smith, of Buffalo, was elected vice-president of the Western New York Alumni Association of the University of Pennsylvania during its foundation meeting held at Buffalo, December 17, 1900.

---

Dr. Julius Ullman, of Buffalo, has been appointed local correspondent of the *Journal of the American Medical Association*—an admirable selection.

---

Dr. Martin J. Downey, of Buffalo, has been appointed assistant surgeon of the Erie railway. The appointment was made on the nomination of Dr. Clayton M. Daniels, chief surgeon.

---

Dr. Sidney A. Dunham read a paper on the Cause and Prevention of Dipsomania before a meeting of the Hornellsville Medical Association at its meeting in December. While in Hornellsville Dr. Dunham was the guest of Dr. Walker at the Steuben Sanitarium.

---

Dr. Daniel Lewis, president, and other members of the State Board of Health, visited Buffalo early last month, and were in official session one day with reference to the nuisance created by the rendering works at East Buffalo.

